# The beauty and the yeast: can the microalgae *Dunaliella* form a borderline lichen with *Hortaea werneckii*?

**DOI:** 10.1007/s13199-020-00697-6

**Published:** 2020-07-27

**Authors:** Lucia Muggia, Polona Zalar, Armando Azua-Bustos, Carlos González-Silva, Martin Grube, Nina Gunde-Cimerman

**Affiliations:** 1 Department of Life Sciences, University of Trieste, via Giorgieri 10, 34127 Trieste, Italy; 2 Department of Biology, Biotechnical Faculty, University of Ljubljana, Večnapot 111, 1000 Ljubljana, Slovenia; 3 Centro de Astrobiología (CSIC-INTA), 28850 Madrid, Torrejón de Ardoz, Spain; 4 Facultad de Ciencias de la Salud, Instituto de Ciencias Biomédicas, Universidad Autónoma de Chile, 8910060 Santiago, Chile; 5 Facultad de Ciencias, Universidad de Tarapacá, Arica, Chile; 6 University of Graz, Institute of Biology, Holteigasse 6, 8010 Graz, Austria

**Keywords:** Atacama Desert, Black yeast, Culture, Halotolerant, Mutualism, Salterns

## Abstract

Lichenized fungi usually develop complex, stratified morphologies through an intricately balanced living together with their algal partners, but several species are known to form only more or less loose associations with algae. These borderline lichens are still little explored although they could inform us about early stages of lichen evolution. We studied the association of the extremely halotolerant fungus *Hortaea werneckii* with the alga *Dunaliella atacamensis*, discovered in a cave in the Atacama Desert (Chile), and with *D*. *salina*, common inhabitant of saltern brines. *D*. *atacamensis* forms small colonies, in which cells of *H*. *werneckii* can be frequently observed, while such interaction has not been observed with *D*. *salina*. As symbiotic interactions between *Dunaliella* and *Hortaea* have not been reported, we performed a series of co-cultivation experiments to inspect whether these species could interact and develop more distinct lichen-like symbiotic structures. We set up co-cultures between axenic strains of *Hortaea werneckii* (isolated both from Mediterranean salterns and from the Atacama cave) and isolates of *D*. *atacamensis* (from the Atacama cave) and *D*. *salina* (isolated from Mediterranean salterns). Although we used different growth media and cultivation approaches, bright field and SEM microscopy analyses did not indicate any mutual effects in these experiments. We discuss the implications for fungal algal interactions along the transition from algal exploiters to lichen symbioses.

## 1 Introduction

Self-supporting symbiotic associations allow organisms to proliferate in habitats where they face limitations to survive by themselves. This ability is well demonstrated by lichens, i.e. symbioses of fungi with algae. The photosynthetic partners are hosted in typically stratified fungal morphologies, which may thrive under extreme environmental conditions. The lichen thalli have a long evolutionary history, dating back to the lower Devonian (Honegger et al. 2013). When and how often this symbiosis emerged has been a matter of phylogenetic studies (e.g. Gargas et al. 1995; Lutzoni et al. 2001; Nelsen et al. 2020), but it is unlikely that the earliest forms of lichens that emerged from fungi already had such an elaborate morphology. To better understand the transition to a lichen-like life style, it would be informative to study their most primitive forms, both by observation and experiment. Such forms, known as borderline lichens, are usually found in marine habitats, with species associations showing a high degree of specialization but without the formation of well-differentiated fungal layers characteristic of true lichens (Kohlmeyer et al. 2004).

**Fig. 1:**
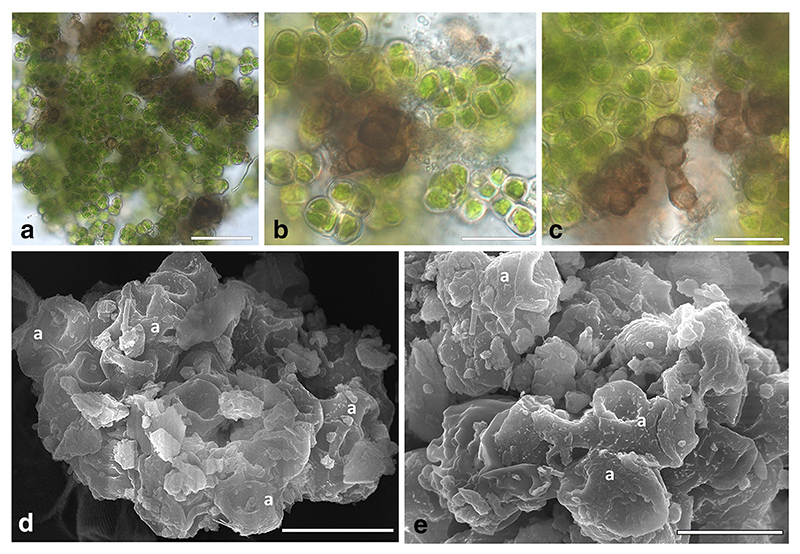
Environmental samples of co-growing *Hortaea werneckii* and *Dunaliella atacamensis*. **a-c.** Squash preparation in water of the algal-fungal clumps: the brownblack *Hortaea* cells can be seen growing in between the algal cells; cells of *D*. *atacamensis* group into tetrads in a palmella state which partially collapse when observed by SEM (D,E). **d, e.** Clumps of algal cells (a) with sand particles, fungal cells in this case are not distinguishable inside the clumps. Scale bars: A) 50 μm; B, C) 20 μm; D, E) 10 μm

Here we report the discovery of an association of fungi and algae, reminiscent of borderline lichens, in a very unusual environment of a cave on the Coastal Range of the Atacama Desert in Chile (Azua-Bustos et al. 2010). As the fog-influenced cave is the habitat of spiders, the silk threads of their webs collect the condensing fog to support the growth of adhering colonies of *Dunaliella atacamensis*. This species is the only member of the unicellular microalgal genus *Dunaliella* reported from a subaerial habitat, where it persists in a palmella-like, colony-like form, with non-motile cells and thick cell wall. The thick cell wall of *D*. *atacamensis* seems to be key for its survival in a subaerial environment, and in this way better tolerate extreme desiccating conditions and provide better structural support (Azua-Bustos et al. 2010, 2012a, b). Interestingly, a closer inspection of the palmella-stage colonies of *D*. *atacamensis* revealed the frequent presence of melanized fungal hyphae (Fig. 1a–c). This fungal component was later identified as the extremely halotolerant black yeast *H*. *werneckii* (Gostinčar et al. 2018; Zalar et al. 2019).

The association of *H*. *werneckii* and *D*. *atacamensis* is similar to that of fungal species of *Saxomyces* and *Lichenothelia* with other green algal lineages (or cyanobacteria), with the latter commonly found on exposed rocks (Muggia et al. 2013; Ametrano et al. 2017, 2019). Such associations are also considered borderline lichens, as the two participating species do not form structures resembling a lichen thallus. In these associations fungal and algal cells grow intertwined with loose contacts observed in both native samples and culture experiments (Ametrano et al. 2017; Muggia et al. 2018). Because these fungi share strongly oligotrophic environments with green algae, they might take advantage from the presence of the primary producers as a first selective advantage to promote lichen-like associations. Interestingly, *Hortaea*, *Lichenothelia* and *Saxomyces* are all members of order Capnodiales, which also includes microfilamentous lichens i.e. *Cystocoleus* and *Racodium* (Muggia et al. 2008). Thus, this order is interesting for studying links between the lichenized and the not lichenized fungal life-styles (Hawksworth 1981; Muggia et al. 2013, 2015; Ametrano et al. 2019).

Extremophilic melanized fungi have an enormous phenotypic plasticity ranging from yeast, mycelial or meristematic stages, and have been isolated in culture frequently, but only a few have been investigated in co-cultures for their interaction with algae (Gorbushina et al. 2005; Brunauer et al. 2007; Ametrano et al. 2017; Muggia et al. 2018). Given our experience co-culturing different species of black fungi and algae, and the observed association between *H*. *werneckii* and *D*. *atacamensis*, we were interested to assess whether the co-culture of *Hortaea werneckii* with species of *Dunaliella* could lead to morphological changes in either species, indicative for mutualistic responses of borderline lichens. Thus, for our experimental approach we also included the species *D*. *salina*, the most widespread species in salterns worldwide and the best adapted to hypersaline and high light conditions. *D*. *salina* cells are flagellate and motile, although under nonoptimal salt concentrations they also form an asexual thickwalled not-motile cyst, referred to as aplanospores or palmella (Wei et al. 2017).

## 2 Materials and methods

### Sampling and characteristics of the species

Environmental samples of *Dunaliella atacamensis* were collected in October 2016 inside a cave located in the Coastal Range of the Atacama Desert (21°15'02.87”S, 70°04'52.33”W). *Hortaea werneckii* was previously isolated from this cave as EXF-6656 (Zalar et al. 2019). The strains *H*. *werneckii* EXF-2000 and *D*. *salina* EXO-4, were isolated from salterns in Sečovlje at the Adriatic coast in 1996 and 2015, respectively. All the fungal and algal strains were available at the culture Microbial Culture Collection Ex of the Infrastructural Centre Mycosmo, MRIC UL, Slovenia (http://www.ex-genebank.com), in the Department of Biology, Biotechnical Faculty, University of Ljubljana (Slovenia).

*Dunaliella* and *Hortaea* are dominant species of algae and fungi found in hypersaline environments, and have been studied in great detail due to their halotolerance, becoming models of salt-adaptation of eukaryotes (Oren 2014; Petrovič et al. 2002; Gostinčar et al. 2011; Plemenitaš et al. 2008, 2014; Gunde-Cimerman et al. 2018; Zalar et al. 2019). *Dunaliella* species can withstand NaCl concentrations ranging from about 0.05 M up to saturation, 5.5 M (Ben-Amotz et al. 2009), its cells are ovoid to pyriform in shape, lack a rigid cell wall and are enclosed by a thin plasma membrane that causes the cells to round up as the external salinity decreases (Oren 2005).

*Hortaea werneckii* is an ascomycetes, black yeast-like fungus characterized by a high morphological polymorphism and physiological plasticity. It is the dominant fugal species in hypersaline waters worldwide (de Hoog 1993; Wollenzien et al. 1995; Sterflinger 2006; Zalar et al. 1999; Gunde-Cimerman et al. 2000), though it was initially known as the etiological agent of the human dermatosis *tinea nigra* (de Hoog and Gerrits van den Ende 1992; Göttlich et al. 1995). *H*. *werneckii* is the only fungus able to grow across the whole range of NaCl concentrations, from 0 to 30% NaCl, with a broad optimum between 6 and 14% NaCl (Butinar et al. 2005; Plemenitaš et al. 2008).

### Isolation and co-cultivation experiment

The Atacama samples consisted of powdery, loose clumps of spiderwebs and dust debris in which algal and fungal colonies were intermixed (Fig. 1). Samples of these clumps were washed twice with distilled water, twice with a 1/10 dilution of Tween80 and finally twice with sterile distilled water to remove dust debris. The washed clumps were then resuspended in sterile water and pipetted on solid agar media. Growth media used were Bold Basal Medium (BBM, Bold 1949) and malt extract agar (MEA), both added with 5% and 10% NaCl. Ten plates per medium were inoculated.

**Fig. 2:**
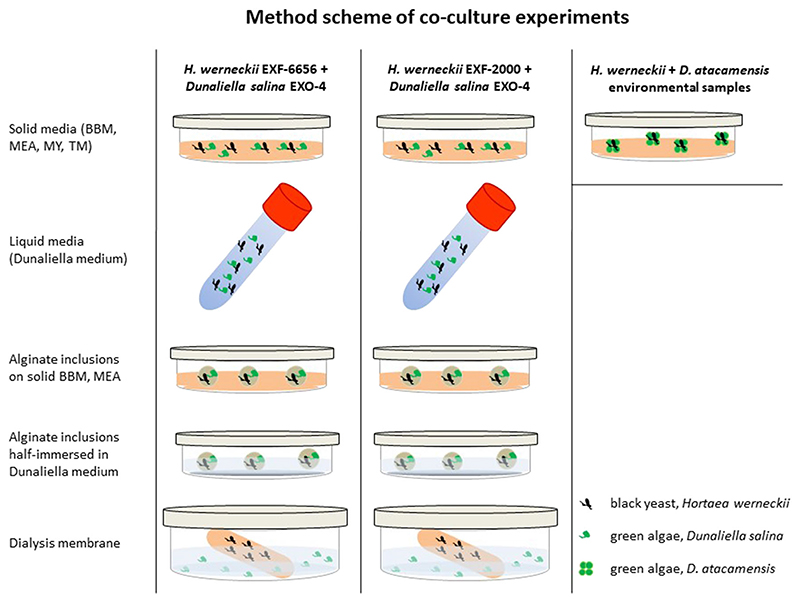
Schematic representation of the co-culture experiments set on solid and in liquid media with the two strains of *Hortaeae werneckii* (EXF-2000, EXF-6656), *Dunaliella salina* and *D*. *atacamensis*

Co-cultivation experiments (Fig. 2) were set using *H*. *werneckii* EXF-6656 previously isolated from Atacama cave samples, the genome sequenced strain *H*. *werneckii* EXF-2000 (Lenassi et al. 2013; Sinha et al. 2017; *Gostinčar* et al. 2018), and *D*. *salina* EXO-4. Co-cultures were set on five liquid and solid media: Bold Basal Medium (BBM), malt extract agar (MEA, Atlas 1995), malt yeast (MY, Lilly and Barnett 1951), the organic rich *Trebouxia*-medium (TM, used for lichen photobionts; Ahmadjian 1967, Muggia et al. 2014, 2018) and *Dunaliella* specific media (Subba Rao 2009). Three types of co-cultures were set 1) solid and liquid media, 2) alginate inclusions, which were either placed on solid media or half immersed in liquid *Dunaliella* medium, and 3) in a dialysis membrane, as detailed below (Fig. 2).

1) Samples of *H*. *werneckii* EXF-6656 and *H*. *werneckii* EXF-2000 were mixed individually with *Dunaliella salina* EXO-4 in 1,5 ml tubes; these mixtures were deposited either on solid media or diluted in the liquid media into 15 ml tubes. Mixed cultures on agar were stored in a growing chamber at 20 °C, with a light-dark regime of 14/10 h with light intensity of 60–100 μmol photons m^−2^ s^−1^ and at a relative humidity of 60%. The tubes with the liquid mix cultures were placed on a shaker to induce the movement of the *Dunaliella* cells.
2) *H*. *werneckii* and *D*. *salina* strains were mixed in an alginate solution following the protocol of Muggia et al. (2018). Alginate inclusions were either deposited on solid MEA and BBM media added with 5% and 10% NaCl, or half immersed in liquid *Dunaliella* medium.
3) *D*. *salina* EXO-04 was grown in *Dunaliella* medium with 15% NaCl, at a cell concentration of 3,6 × 10^5^ cells/ml, while *H*. *weneckii* strains EXF-2000 and EXF-6656 were grown in liquid yeast nitrogen based (YNB) medium at 5% NaCl, at a concentration of 5,1 × 10^5^ cells/ml. 500 ml of MEA 10% NaCl with 0.5% agar were prepared to maintain the medium semi-solid. Ten dialysis membranes were filled with this medium. Membranes were previously cut in pieces of about 15–20 cm, soaked in sterile distilled water to be softened, and then filled with 20 ml of the MEA 10% NaCl medium. The membranes thus prepared were then inoculated with 0,7 ml of *H*. *weneckii* strains EXF-2000 and EXF-6656, five with each *Hortaea* strain respectively. Dunaliella-media with 15%, 20% and 25% NaCl concentrations were prepared and sterilized. 10 ml of *D*. *salina* culture suspensions were then inoculated in 190 ml of each of these media. These solutions were then poured into glass dishes, and dialysis membranes inoculated with *Hortaea* cells immersed in them. Co-cultures were then covered with glass caps, sealed with parafilm, stored at room temperature under natural light on the laboratory bench and shaken daily by hand for about 1 month.

### Microscopic analyses

Bright field microscopy was used on environmental samples and isolates using a Zeiss light microscope mounting samples in water and acquiring digital pictures with a ZeissAxioCam MRc5 digital camera fitted to stereo and light microscopes. These microphotographs were digitally optimized using Combine ZM software (image processing software available at www.hadleyweb.pwp.blueyonder.co.uk/CZM/). Scanning electron microscopy (SEM, Quanta250 SEM, FEI, Oregon, USA) was performed for environmental samples from Atacama using both the e-SEM function and the traditional gold sputtering (Muggia et al. 2011).

## 3 Results and discussion

**Fig. 3:**
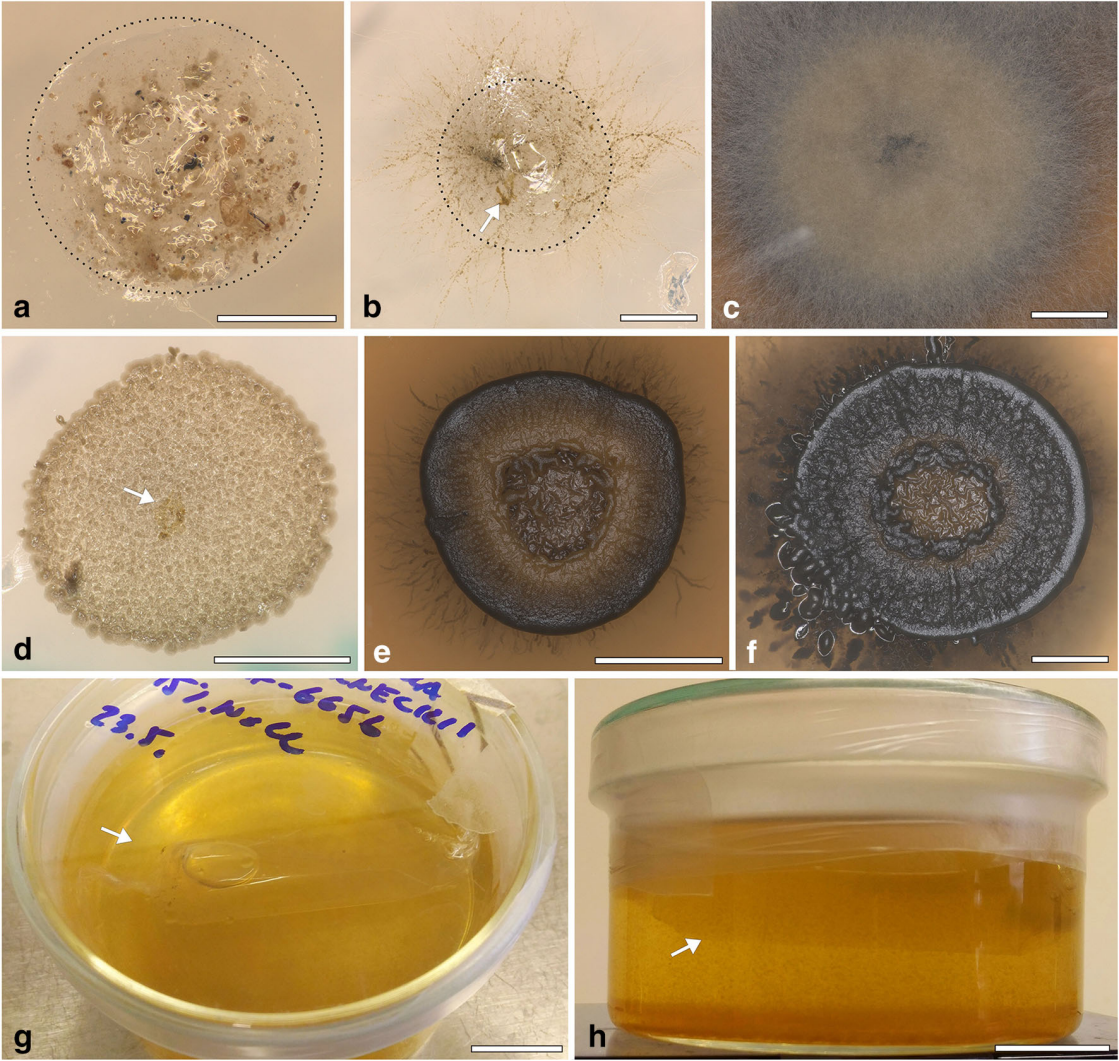
Coculture experiments of *Hortaea werneckii* and *Dunaliella* spp. **a.** Alginate inclusion (delineated by dot line) on BBM 10 M NaCl used to attempt isolation and cogrowth of *H*. *werneckii* and *D*. *atacamensis* from Atacama environmental sample. Soil debris and fungal + algae clumps are visible. **b, c.** Alginate inclusion (delineated by dot line) on MEA 10%NaClto attempt cogrowth of *H*. *werneckii* EXF-6656 (black hyphae) and *D*. *salina* (arrow) as observed after 1 week (**b**) and after 1 month (**c**). Note how *H*. *werneckii* completely overgrew the inoculum in this last case. **d-f.** Alginate inclusions on MEA 5% NaCl to attempt cogrowth of *H*. *werneckii* EXF-2000 (black cells in D, yeast colony in E and F) and *D*. *salina* (arrow), 1 week (D), 3 weeks (E) and 5 weeks (F) after inoculation. Note how *H*. *werneckii* completely overgrew the inoculum in this last case, and even addition digested the alginate used. **g, h.** Dialysis membrane filled with MEA medium (arrow) and inoculated with *H*. *werneckii*, submersed in *Dunaliella* medium. Scale bars: A, B) 1 mm; C) 4 mm; D, F) 2 mm; E) 5 mm; G, H) 2,5 mm

**Fig. 4:**
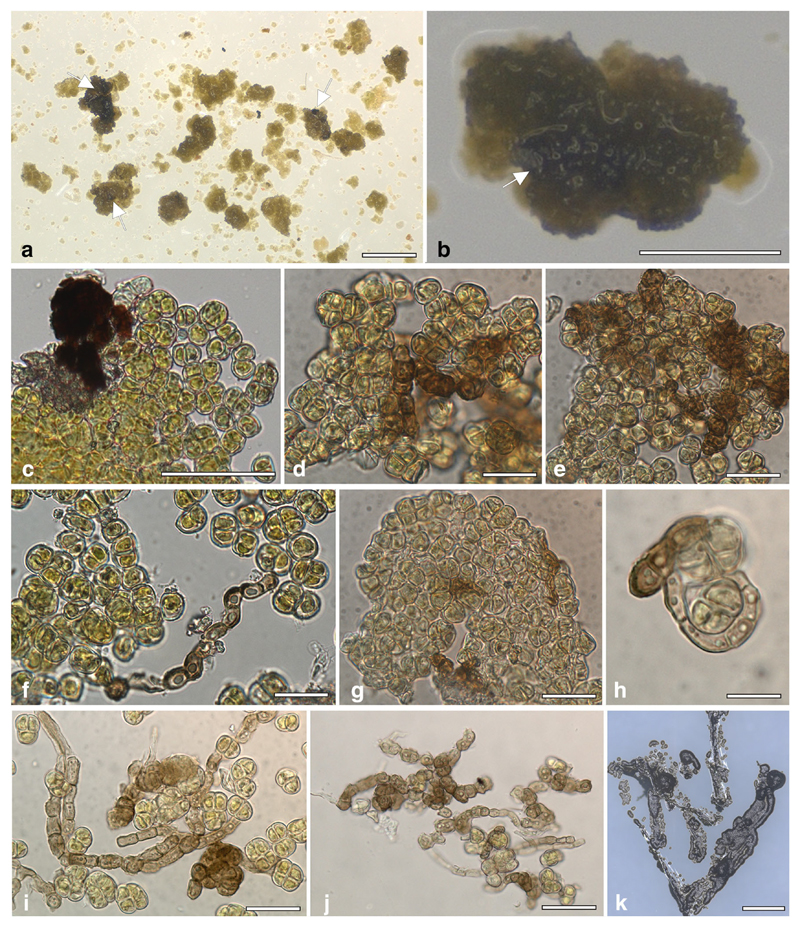
Isolation of *Hortaea werneckii* and *Dunaliella atacamensis* environmental samples on solid medium MEA 5%NaCl. **A, B.** Algal clumps enwrapping fungal cells (white arrows) were inoculated axenically on agar medium. **C-J.** Squash preparation in water of the algal-fungal clumps after 1 month of inoculation. Black *Hortaea* cells can be observed growing in between the algal tetrads. In the rare cases where *Hortaea* develop filamentous hyphae, these enfold the algal tetrads (H). **K.** *Hortaea werneckii* EXF-6656 isolated from environmental samples of the Atacama cave. Scale bars: A) 1 mm; B) 250 μm; C) 100 μm; D-G, I, J) 20 μm; I) 10 μm; K) 1 mm

Despite several attempts in two separate laboratories, it was not possible to grow *D*. *atacamensis* in vitro. The isolation of *D*. *atacamensis* was repeatedly attempted using different approaches, either directly plating the samples on growth media or including them in alginate (Figs. 2, 3). Different culture media were prepared with various NaCl concentrations in order to reproduce the saline conditions of its natural environment, or by using various media for algal growth, including a *Dunaliella* specific medium and protein rich media, i.e. MY and MEA, to take into account the protein-rich spider webs (Vollrath 2000; Römer and Scheibel 2008) where *D*. *atacamensis* and *H*. *werneckii* were found. To test whether *D*. *atacamensis* could require a critical metabolite produced by *H*. *werneckii*, its co-cultivation was also attempted, but this also proved unsuccessful. Initiating the algal growth alone does not seem to depend on the presence of *H*. *werneckii*, as its cells were clearly present and visibly entangled among the algal cells (Fig. 4). Interestingly, the fungus continued to grow mostly in its yeast form (with only a few hyphae observed) and forming small cell clumps detectable as black spots immersed in the algal colonies (Fig. 4). Most likely, a specific abiotic or biotic factor is lacking explaining the failed proliferation of *D*. *atacamensis* in vitro. Among other potential explanations, the washing steps performed in order to remove dust particulate might have removed critical nutrients essential for the life cycle of this peculiar alga. Also, in vitro culture plates were incubated in a chamber with a relative humidity set at 60%, which might be too dry for *D*. *atacamensis* to divide actively. Another explanation is that, at the palmella stage (when *Dunaliella* cells become more rounded and embedded in a layer of exopolysaccharides), it only proliferates under fluctuating hygroscopic conditions of the subaerial native habitat, conditions difficult to reproduce in the lab. Despite the lack of active growth, algal cells on the media used remained green and viable for several months with or without *H*. *werneckii* (Fig. 4). Only later the algal cells started to bleach, with the darker *Hortaea* cells becoming evident in the case of co-cultures. Interestingly, *Hortaea* cells grew inside algal cell clumps, which explains the difficulty to detect them by SEM inspection (Fig. 1d, e). Although no viable and stable coculture could be obtained with this approach, it is still of interest to discuss this result in a broader framework of borderline lichens further below.

Similarly, culture experiments with *H*. *werneckii* and *Dunaliella salina* did not showed clear signs of fungal-algal association. Here, *D*. *salina* was first able to grow exponentially in its motile stage, later entering a stationary phase in which cells started to produce orange/red carotenoids (reported as a stress marker; Teodoresco 1905, Faraloni and Torzillo 2017). These cells then formed clumps that could be indicative of the palmella stage, also an indication of stress in *Dunaliella* species. In turn, *H*. *werneckii* showed a much faster growth rate with no visible inhibitions. When *D*. *salina* and *H*. *werneckii* were co-cultured on solid medium, or in the alginate inclusions, despite a relative ratio of *Hortaea/Dunaliella* cells much lower than one, *Dunaliella* cells were completely overgrown by the fungal cells within three to 4 weeks (Fig. 3). To test a potential chemotactic response towards metabolites produced by *H*. *werneckii* or *Dunaliella* cells, we also attempted separating the cultures with a dialysis membrane. However, *D*. *salina* in this system grew at very slow rates in the liquid media used, while *Hortaea* grew poorly in the solid medium inside the membrane, growing instead in the portions of the membrane emerging from the liquid medium, suggesting a preference for a higher oxygen access.

Intrinsic characteristics of *Dunaliella* cells may have also hindered potential contact events with fungal cells. As aforementioned, although *D*. *salina* is a motile species with an exposed membrane, *D*. *atacamensis* grows instead as tight clumps of immobile cells which are coated by a thick layer of EPS that may hamper the formation of contacts with *Hortaea* cells. Furthermore, melanization makes fungal cell walls more rigid and less flexible for tight contacts with algae, and only very few filamentous hyphal elements are found beside yeast cells in the algal clumps. Special interactive structures such as haustoria are also missing when other melanized rock-inhabiting fungi grow together with unicellular algae either in nature or in vitro (i.g. species of *Lichenothelia* and *Saxomyces*; Muggia et al. 2013, 2018; Ametrano et al. 2017).

Fungal species able to establish lichens or ‘lichen-like’ symbiosis have evolved in divergent lineages within the Dothideomycetes (Schoch et al. 2009). Examples of rather primitive thallus structures are *Cystocoleus ebeneus* and *Racodium rupestre* (Capnodiales). The fungi develop a tight fungal coat of one cell layer thickness around the filamentous photobiont (a thread of *Trentepohlia*), which does not differ much from free-living forms and determines the shape of the thallus (Muggia et al. 2008). These species may nonetheless be representative for well-established lichen symbioses.

Other rock-inhabiting melanized fungi and *Hortaea* in the order Capnodiales generally share traits of stress tolerance allowing them to survive in oligotrophic and extremely dry environments. Species of *Lichenothelia* and *Saxomyces* do not form any clear thallus structures, but frequently associate with coccoid green algae, and may also form phenotypically more plastic mycelia (with filamentous or short yeast-like cells; Muggia et al. 2015). Their association with microscopic algae potentially improves the meager carbon supplies of melanized oligotrophic fungi (Gostinčar et al. 2012). Interestingly, the genus *Lichenothelia* also includes some species living more or less specifically on lichens as hosts (Kocourková and Knudsen 2009), suggesting that they may take direct or indirect benefit of the host’s algal productivity.

Mycophycobioses are formed by fungi immersed in unchanged colonies of algae but without any sign of structural integration. But necessarily, in some beneficial case such interactions may support the production of sexual fruitbodies by the algicolous fungi. With increased integration of the fungal-algal interaction and higher specialisation, the criteria of a broderline lichen are thus fulfilled (Kohlmeyer et al. 2004). From there, a stepwise transition may have lead to typical lichen symbioses as self-sustaining ecosystems (Hawksworth and Grube 2020), where fungi usually represent the exhabitant partner forming a covering structure of hyphal cells embedded in extracellular polysaccharides (Grube and Wedin 2016; Spribille et al. 2020). Recent findings showed that the evolutionary success of lichen symbioses may be assisted by the interplay of additional symbionts, (photosynthetic algae, bacteria and yeasts) that co-inhabit the lichen thalli, and potentially contribute to their structural integrity (Spribille et al. 2020). Thus, the formation of a lichen thallus might be triggered by yet unidentified additional players, a hypothesis that goes further beyond the ‘simplistic’ affinity between a fungus and an algae. If this would be the case of the association between *H*. *werneckii* and *D*. *atacamensis*, the washing steps used in our methods might have eliminated or reduced the presence of potential lichen-enhancing microorganisms.

Although *Hortaea* and *Dunaliella* have been found together and no mutual benefits or interactions could be proved so far using the current approaches, it canno be dismissed that their association may still be in a very early stage along the transition towards lichens, or that more stressful conditions should have been tested. However, this does not mean that lichen-like associations necessarily emerged out of damp and sheltered places. It remains nevertheless interesting to include this species in comparative genomics approaches to assess whether specific genomic arrangements correspond to the transition from substrate-immersed fungi to the exhabitant lichen fungi, or whether these genomic patterns are secondary factors for the evolution of lichens. In this context, extreme environments merit further exploration as biodiversity hotspots of microorganisms with the yet to be discovered potential to form new symbiotic associations.
